# Intersecting Sex Work and Substance Use Risk Among Sexual and Gender Minoritized Individuals Recruited Online in San Francisco, California: Survey Results

**DOI:** 10.2196/83555

**Published:** 2026-02-11

**Authors:** Sean Arayasirikul, Jarett Maycott, Angela Olivares

**Affiliations:** 1 The Legacy Center Joe C Wen School of Population & Public Health University of California, Irvine Irvine, CA United States; 2 San Francisco Department of Public Health San Francisco, CA United States

**Keywords:** sex work, substance use, domestic violence, intimate partner violence, online recruitment, sexual and gender minoritized community health, social media

## Abstract

This research letter examines sex work and substance use associations in a sample of sexual and gender minoritized individuals recruited online in San Francisco, California. This study found that a history of sex work was prevalent and that people with a history of sex work were more likely to recently report using controlled substances and experience domestic violence.

## Introduction

Sexual and gender minoritized (SGM) communities experience victimization because of structural oppression [1]. The confluence of social exclusion, economic hardship, and the internet supports the formation of a sex work economy [2,3]. People in sex work experience health inequities due to their unique occupational health exposures [4]. Yet there is little research on online help-seeking SGM populations [5]. We address this gap and characterize the prevalence of sex work history among SGM individuals seeking help online and their sexual and substance use–related prevention and treatment needs.

## Methods

### Ethical Considerations

The study was approved by the University of California, San Francisco institutional review board (20-33169). Participants provided informed consent, could opt out anytime, and received US $30. Data were deidentified to protect participants’ privacy and confidentiality.

### Study Design and Recruitment

This is a cross-sectional analysis of 409 people recruited online in San Francisco by using social medial advertisements on Facebook, Instagram, and Grindr in 2022-2024. Advertisements sought out potential participants seeking help for substance use, mental health, and HIV. Once they clicked on the advertisement and were directed to a study interest website, they were contacted by staff to screen for eligibility, informed consent was obtained electronically, and they completed a baseline assessment. Eligibility criteria included aged 18 years or older, seeking help for substance use prevention/treatment or related health topics (eg, HIV, mental health), live in San Francisco, identify as a man who had sex with men or a trans woman, and had smartphone access.

### Measures and Analysis

We analyzed the following demographics: age, race/ethnicity, gender identity, sexual orientation, HIV status, housing stability, and socioeconomic status. Recent substance use was measured by asking how many days in the last 30 days, if any, did they use each of the following substances: tobacco, vaping products, binge drinking (having 5 or more alcoholic beverages at the same time or within a couple of hours of each other for people assigned male sex at birth), marijuana, prescription opioids, nonprescription opioids, other prescription drugs, illicit drugs (crack/cocaine, amphetamine/methamphetamine, hallucinogens, inhalants), and injection drug use [6]. Responses were recoded dichotomously for any recent use (yes/no). We measured participants’ history of sex work if they had ever had sex with someone in exchange for money, drugs, or shelter (yes/no) [6]. We assessed participants’ risk perception of harm when engaging in condomless sex and sex while using drugs or alcohol. Responses were dichotomized into low risk (no risk, slight risk, unknown risk) and high risk (moderate risk, great risk). Self-efficacy to refuse condomless sex was assessed by participants’ agreement with the statement, “I could refuse if someone wanted to have sex without a condom or dental dam” [6]. Responses were dichotomized into agree (strongly agree, agree) and disagree (disagree, strongly disagree). Domestic violence was measured by asking participants if anyone with whom they had an intimate relationship had emotionally, physically, or sexually abused them in the last 3 months (yes/no) [6]. STATA version 17 was used to create logistic regression models to test associations between history of sex work and recent substance use and sexual risk outcomes, adjusted for age, gender identity, race/ethnicity, socioeconomic status, and housing stability.

## Results

Many participants (168/409, 41.08%) reported a history of sex work. The most frequently reported in the last 30 days was illicit drugs (227/409, 55.50%) followed by marijuana (220/409, 53.79%), binge drinking (160/409, 39.12%), and tobacco (155/409, 37.90%). A majority of participants perceived condomless sex (287/409, 70.17%) and sex while using drugs or alcohol (349/409, 85.33%) as high risk and could refuse condomless sex (361/409, 88.26%). Almost 22.98% (94/409) had experienced domestic violence in the last 3 months (Table 1).

**Table 1:** Sample characteristics, recent substance use, sexual risk perception, and self-efficacy to refuse condomless sex and behaviors among sexual and gender minoritized people recruited online in San Francisco, California, 2022-2024 (N=409).

				Overall (N=409), n (%)		Sex work status		
						Yes (n=168), n (%)		No (n=241), n (%)
**Demographics**								
	**Age (y)**							
		18-29	103 (25.18)		31 (18.45)		72 (29.88)	
		30-39	112 (27.38)		54 (32.14)		58 (24.07)	
		40-49	77 (18.83)		34 (20.24)		43 (17.84)	
		50+	117 (28.61)		49 (29.17)		68 (28.22)	
	**Race/ethnicity**							
		White	169 (41.32)		71 (42.26)		98 (40.66)	
		Latino/a/x/e	113 (27.63)		51 (30.36)		62 (25.73)	
		Asian, Pacific Islander, and Native Hawaiian	50 (12.22)		13 (7.74)		37 (15.35)	
		Black	38 (9.29)		16 (9.52)		22 (9.13)	
		More than one or other	39 (9.54)		17 (10.12)		22 (9.13)	
	**Gender identity**							
		Cisgender man	327 (79.95)		123 (73.21)		204 (84.65)	
		Transgender woman or gender expansive	82 (20.05)		45 (26.79)		37 (15.35)	
	**Sexual orientation**							
		Bisexual	47 (11.49)		22 (13.10)		25 (10.37)	
		Gay/lesbian	282 (68.95)		105 (62.50)		177 (73.44)	
		Other	48 (11.74)		22 (13.10)		26 (10.79)	
		Straight/heterosexual	32 (7.82)		19 (11.31)		13 (5.39)	
	**HIV status**							
		Not a person with HIV	278 (67.97)		96 (57.14)		182 (75.52)	
		Person with HIV	131 (32.03)		72 (42.86)		59 (24.48)	
	**Housing stability**							
		Stable	337 (82.40)		123 (73.21)		214 (88.80)	
		Unstable	72 (17.60)		45 (26.79)		27 (11.20)	
	**Socioeconomic status**							
		Above federal poverty line	338 (82.64)		124 (73.81)		214 (88.80)	
		Below federal poverty line	71 (17.36)		44 (26.19)		27 (11.20)	
**Recent substance use (last 30 days)**								
	**Used tobacco**							
		No	254 (62.10)		81 (48.21)		173 (71.78)	
		Yes	155 (37.90)		87 (51.79)		68 (28.22)	
	**Used vaping products**							
		No	288 (70.42)		109 (64.88)		179 (74.27)	
		Yes	121 (29.58)		59 (35.12)		62 (25.73)	
	**Binge drinking**							
		No	249 (60.88)		116 (69.05)		133 (55.19)	
		Yes	160 (39.12)		52 (30.95)		108 (44.81)	
	**Used marijuana**							
		No	189 (46.21)		77 (45.83)		112 (46.47)	
		Yes	220 (53.79)		91 (54.17)		129 (53.53)	
	**Used prescription opioid drugs**							
		No	398 (97.31)		160 (95.24)		238 (98.76)	
		Yes	11 (2.69)		8 (4.76)		3 (1.24)	
	**Used nonprescription opioid drugs**							
		No	396 (96.82)		159 (94.64)		237 (98.34)	
		Yes	13 (3.18)		9 (5.36)		4 (1.66)	
	**Used other prescription drugs**							
		No	361 (88.26)		141 (83.93)		220 (91.29)	
		Yes	48 (11.74)		27 (16.07)		21 (8.71)	
	**Used illicit drugs**							
		No	182 (44.50)		56 (33.33)		126 (52.28)	
		Yes	227 (55.50)		112 (66.67)		115 (47.72)	
	**Injected drugs**							
		No	366 (89.49)		135 (80.36)		231 (95.85)	
		Yes	43 (10.51)		33 (19.64)		10 (4.15)	
**Sexual risk perception, efficacy and behaviors**								
	**Condomless sex**							
		High risk	287 (70.17)		122 (72.62)		165 (68.46)	
		Low risk	122 (29.83)		46 (27.38)		76 (31.54)	
	**Sex while using drugs or alcohol**							
		High risk	349 (85.33)		142 (84.52)		207 (85.89)	
		Low risk	60 (14.67)		26 (15.48)		34 (14.11)	
	**Self-efficacy to refuse condomless sex**							
		Disagree	48 (11.74)		25 (14.88)		23 (9.54)	
		Agree	361 (88.26)		143 (8.12)		218 (90.46)	
	**Domestic violence (past 3 months**)							
		No	315 (77.02)		102 (60.71)		213 (88.38)	
		Yes	94 (22.98)		66 (39.29)		28 (11.62)	

**Figure 1:**
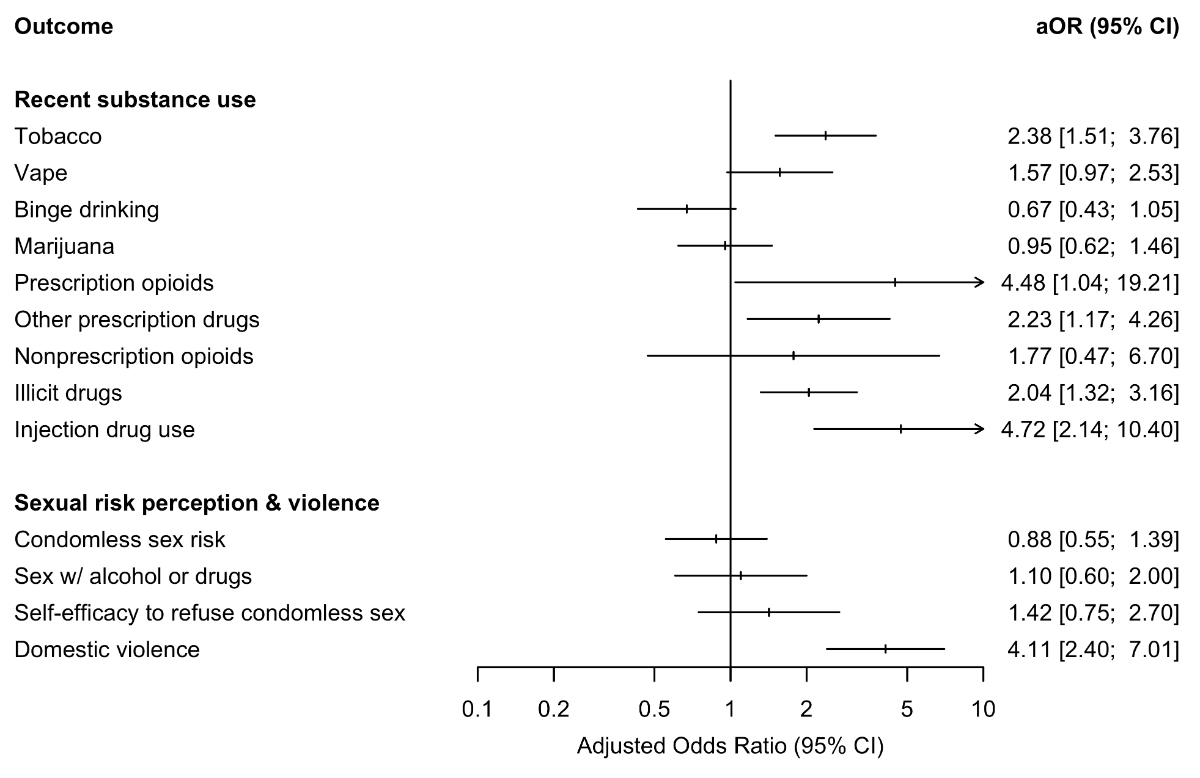
Associations between history of sex work and recent substance use and between sexual risk perception and violence among sexual and gender minoritized people recruited online in San Francisco, California, 2022-2024 (N=409). aOR: adjusted odds ratio.

Adjusting for potential confounders, people with a history of sex work (Figure 1) were more likely to report using tobacco (adjusted odds ratio [aOR] 2.38, 95% CI 1.51-3.76), prescription opioid drugs (aOR 4.48, 95% CI 1.04-19.21), other prescription drugs (aOR 2.23, 95% CI 1.17-4.26), illicit drugs (aOR 2.04, 95% CI 1.32-3.16), and have injected drugs (aOR 4.72, 95% CI 2.14-10.40) compared to people with no sex work history. People with a history of sex work had 4.11 times the odds of experiencing domestic violence recently compared to counterparts with no sex work history (95% CI 2.40-7.01). No statistically significant association was observed between history of sex work and self-efficacy to refuse condomless sex.

## Discussion

We found that SGM participants with a history of sex work were more likely to report recent use of controlled substances. Although no statistically significant associations exist between history of sex work and sexual risk perception level and self-efficacy to refuse condom use, our findings indicate that domestic violence may be heightened for SGM people with a history of sex work. SGM populations report similar or greater rates of domestic or intimate partner violence compared to their non-SGM counterparts [7], and intimate partner violence is a common experience among people who engage in sex work [8]. Relationship dynamics among SGM partnerships can vary in composition with relationship to sexual and gender roles, power, and how violence is enacted and experienced. A recent systematic literature review found that bidirectional violence was the most common among SGM intimate partners compared to non-SGM partners [9]. The interplay between substance use, violence, and trauma are complex and must be explored in future [10]. This study has limited generalizability because of its design and possible sampling bias toward people seeking help online who may be experiencing heightened risk. Despite this, these findings reinforce the need for substance use prevention efforts to serve people with a history of sex work and address domestic violence as a unique violence exposure. Although stigma remains a significant barrier for people engaged in sex work and those using substances, addressing domestic violence may offer a whole-person alternative to intervene on the material impacts of violence and substance use behaviors that may be both enabling and coping mechanisms of violence. Public health interventions that cross-train substance use providers about domestic violence and sex work literacy, and conversely, are needed to better facilitate screening, referral, and treatment.

## Abbreviations

aOR: adjusted odds ratio
SGM: sexual and gender minoritized

## Data Availability

The datasets generated or analyzed during this study are not publicly available as they contain details that could be used to identify participants but are available from the corresponding author on reasonable request.
